# Experiences of Healthcare Professionals in a Street Clinic in a Municipality in Southern Brazil

**DOI:** 10.3390/ijerph23050601

**Published:** 2026-05-01

**Authors:** George Antônio dos Santos Júnior, Lucas Hoffmann Dias, Tamara Tomitan Richter, Jeferson Luis Lima da Silva, Tânia Maria Gomes da Silva

**Affiliations:** 1 School of Medicine, Cesumar University, Maringá 87050-390, PR, Brazil; georgejunior28@hotmail.com (G.A.d.S.J.); lucashoffmann4126@gmail.com (L.H.D.); 2 Instituto Cesumar de Ciência, Tecnologia e Inovação (ICETI), Cesumar University, Maringá 87050-390, PR, Brazil; tamara.richter@hotmail.com (T.T.R.); profjefersonlima@outlook.com (J.L.L.d.S.); 3 Graduate Program in Health Promotion, Cesumar University, Maringá 87050-390, PR, Brazil

**Keywords:** street clinic, homeless population, healthcare professionals, primary health care, Brazil

## Abstract

**Highlights:**

**Relevance to Public Health—How does this work relate to a public health problem?**

- The Street Clinic (Consultório na Rua—CnR) is a strategic component of Primary Health Care aimed at populations experiencing homelessness, a group characterized by high levels of social and health vulnerability in Brazil and globally.
- Despite normative advances, the CnR operates under chronic underfunding, institutional instability, and precarious employment arrangements, which hinder the full implementation of the policy.

**Significance for Public Health—Why is this work significant for public health?**

- This study foregrounds the perspective of healthcare professionals, revealing how ethical suffering and creative resistance coexist within street-based practice.
- The hermeneutic analysis highlights the gap between policy design and the concrete realities of practice, contributing to the scientific understanding of equity in access to healthcare.

**Implications for Public Health—What are the main implications for professionals, managers, and researchers?**

- Strengthening the CnR requires stable employment contracts, continuous emotional support for teams, and effective integration with housing and social assistance policies.
- The CnR must be recognized as a state policy, with institutionalized care pathways, adequate infrastructure, and continuing education strategies for professionals.

**Abstract:**

The Street Clinic (Consultório na Rua—CnR) is a strategic component of Primary Health Care in Brazil, aimed at populations experiencing homelessness, a group characterized by high levels of social and health vulnerability. This study critically analyzes the experiences of healthcare professionals working within a CnR team, identifying the meanings attributed to their work, the challenges encountered, and the strategies developed within the territory. This is an exploratory study with a qualitative approach, grounded in health narratives and the philosophical hermeneutics of Hans-Georg Gadamer. Four professionals participated, representing the totality of eligible members of a team in a medium-sized municipality in Southern Brazil, with between one and eleven years of experience in the service. Hermeneutic analysis revealed that the CnR functions as an entry point to Primary Health Care and Psychosocial Care, with the bond between team and users serving as the primary mechanism for overcoming barriers to access. Professionals report ethical suffering arising from the tension between their commitment to comprehensive care and the structural limitations of the service, including shortages of supplies, institutional instability, and precarious employment arrangements. It is concluded that strengthening the CnR requires not only investment in infrastructure and expansion of the teams, but also policies that recognize and support the complexity of street-based work, including care for the caregivers themselves.

## 1. Introduction

The population experiencing homelessness is characterized by profound vulnerabilities that cannot be explained solely from an individual perspective, but rather as the result of interconnected social and programmatic factors, exacerbated by the neoliberal capitalist model, which intensifies poverty, the erosion of rights, the expansion of informal settlements, labor precarization, and housing deprivation [1,2].

In the context of the structural crisis of capital, some individuals are left with no alternative but to occupy public spaces or degraded areas, exposed to cold, rain, poor hygiene conditions, nutritional deprivation, and various forms of violence—sexual, physical, and psychological—including social invisibility [3]. These conditions violate human rights, compromise overall health, and undermine the principle of equity proposed by the Brazilian Unified Health System (Sistema Único de Saúde—SUS) [4].

In 2024, the United States reported 771,480 individuals experiencing homelessness, and a study following more than 140,000 registered homeless individuals demonstrated elevated mortality rates within this population [5]. Furthermore, 2024 saw an 18% increase compared to 2023, associated with the housing crisis, inflation, and the discontinuation of post-COVID prevention programs [6]. In the city of Paris, between 2014 and 2023, 1559 deaths were recorded among this population, with 75.9% occurring in individuals under 65 years of age [7].

In Brazil, this phenomenon has grown at a faster rate than the general population: 211% between 2012 and 2022, compared to an 11% population increase in the same period [8]. Despite the legal framework established by the National Policy for the Homeless Population, as of March 2025, the Cadastro Único recorded 335,151 individuals living under these conditions [9]. In the municipality where the present study is conducted, among 975 registered individuals, 953 report no contact with family members. The street constitutes the most frequent sleeping location, with 655 records, followed by shelters, with 335 [9,10].

The Street Clinic (Consultório na Rua—CnR), a strategic policy within Primary Health Care integrated into the Psychosocial Care Network (Rede de Atenção Psicossocial—RAPS), emerges as an institutional response to the health needs of the homeless population, providing comprehensive and non-judgmental care through mobile teams operating in public squares, parks, shelters, and Social Assistance Reference Centers (Centro POP), delivering services ranging from wound care and psychological support to social reintegration initiatives [11,12,13,14]. Despite its relevance, CnR work faces significant structural challenges, including lack of infrastructure, territorial violence, and difficulties in maintaining care bonds [15,16].

Although Street Clinic services are expanding in Brazil, research examining the implementation of this policy from the perspective of the professionals who carry it out on a daily basis remains limited. This gap is relevant because the enactment of public policies depends, to a great extent, on how frontline workers interpret, negotiate, and adapt institutional guidelines within contexts marked by structural constraints. In the case of the Street Clinic, understanding these experiences helps illuminate the persistent tension between the ideals of the policy and the concrete conditions of its implementation in the territories.

In this context, the present study is guided by the following question: how do Street Clinic professionals experience the tension between their ethical commitment to care and the structural limitations that shape their daily work? The objective is to critically analyze the experiences of a Street Clinic team regarding policy implementation and the self-assessment of care, identifying the meanings attributed to their work, the challenges encountered, and the strategies developed within the territory.

## 2. Materials and Methods

### 2.1. Study Design

This is an exploratory study with a qualitative approach, grounded in the theoretical and methodological assumptions of health narratives within the framework of the philosophical hermeneutics of Hans-Georg Gadamer [17]. It thus constitutes a dialogical encounter between subjects—researcher and participant—which, rather than merely scientifically verifying the implementation of clinical protocols, proposes a “living dialogue” [17] that seeks, through the folds of language, to understand how healthcare professionals enact care practices for individuals living under conditions of extreme vulnerability. Drawing on the critical pedagogy of Paulo Freire and the philosophical ontology of Paul Ricoeur, oral narratives were employed as a strategy to access the subjective and symbolic dimensions of work within the CnR, capturing emotions, contradictions, and meanings that do not emerge in formal records [18,19].

We selected hermeneutics as our philosophical framework over other methodologies that examine lived experience, such as phenomenology, because our inquiry requires understanding the Street Clinic not in isolation but within its institutional, political, and structural contexts. While phenomenological approaches seek to bracket contextual factors and describe the essential structures of experience, hermeneutics is fundamentally concerned with how meaning emerges through interpretation within these contexts. The Street Clinic professionals’ experiences are inseparable from the structural constraints, policy tensions, and institutional realities they navigate daily. Hermeneutic analysis thus illuminates how these professionals construct meaning through their ongoing interpretation of—and negotiation with—these constitutive contextual forces.

### 2.2. Participants

Participant selection followed previously established inclusion criteria: employment within the CnR for a period equal to or greater than six months and formal training in a health-related field. Team members without a healthcare background were excluded. Applying these criteria to the CnR team in Maringá, composed of six professionals, the four participants represented the totality of eligible individuals. In qualitative research grounded in philosophical hermeneutics, the criterion of sufficiency is not numerical but interpretive [19,20], with the adequacy of the corpus assessed through thematic saturation.

### 2.3. Data Collection

Individual, semi-structured interviews were conducted in two separate stages between April and May 2025. In the first meeting, the research objectives were presented, ethical procedures were explained, and written informed consent was obtained. Participants were assured of anonymity through the use of alphanumeric codes (PS1–PS4), and were informed of their right to withdraw at any point without consequences. The second stage consisted of the recorded interview itself, guided by a flexible interview guide with open-ended questions designed to elicit narratives about: (1) meanings attributed to work in the CnR; (2) daily challenges encountered; (3) coping strategies and professional practices; and (4) perceived gaps between policy and practice. Interviews lasted between 45 and 75 min. All interviews were audio-recorded with participant consent and subsequently transcribed verbatim by the research team. Transcripts were returned to participants for member-checking, allowing them to review, clarify, or expand their statements.

### 2.4. Data Analysis

Data analysis was operationalized in three stages. First, the researchers formally articulated their pre-understandings, acknowledging their evaluative positions regarding the homeless population, the Brazilian Unified Health System (SUS), and work in collective health—an essential condition for enabling interpretive displacement rather than mere confirmation of prior expectations. Second, an initial open reading of the transcripts was conducted, followed by iterative readings guided by the hermeneutic circle [17], wherein each statement was interpreted in light of the entire interview, each interview in relation to the full corpus, and the corpus in dialogue with the institutional and territorial context of the service. Third, thematic axes emerged inductively through iterative coding. Two independent researchers (G.A.S.J. and L.H.D.) conducted open coding of the transcripts, identifying meaningful units of analysis within the participants’ narratives. These codes were then discussed and organized into preliminary thematic groupings. Through successive rounds of reading and discussion, guided by the hermeneutic circle, these groupings were refined and consolidated into seven thematic axes: (1) Social Vulnerability; (2) Bond with Users; (3) Infrastructure and Resources; (4) Human Resources and Organization; (5) Communication with Users; (6) Therapeutic Planning; and (7) Notable/Negative Situations, reflecting professionals’ emotional responses and coping mechanisms.

Importantly, the hermeneutic ‘dialogue’ central to this philosophical approach occurs through this iterative analytical process itself—not merely through the presentation of verbatim participant quotations, but through the sustained engagement between researchers and text, the consensus-building between independent coders, and the circular movement between interpretive parts and the meaningful whole. This process of iterative coding and thematic refinement constitutes the dialogical encounter through which meaning emerges and is understood within the context of the Street Clinic’s structural realities.

### 2.5. Ethical Considerations

The research project was approved by the Research Ethics Committee of Universidade Cesumar, Maringá, Brazil (Opinion No. 7497004, April 2025). The Municipal Health Secretariat also issued formal approval, ensuring the institutional legitimacy required to conduct the study within public health services.

## 3. Results and Discussion

The presentation of results is grounded in the understanding that qualitative analysis requires attention both to the objective elements that constitute participants’ profiles and to the subjective dimensions expressed in their narratives. The findings are organized to articulate the sociodemographic characteristics of the professionals, the meanings they attribute to their work within the CnR, and the structural conditions that shape their practices.

Initially, as presented in Table 1, the sociodemographic characterization of participants allows for an understanding of the composition and internal dynamics of the investigated Street Clinic team. To preserve anonymity, professionals were identified using alphanumeric codes.

The multiprofessional composition presented in Table 1 reflects central guidelines for the functioning of Street Clinic teams, as established by the Brazilian Ministry of Health [11,21]. The presence of professionals from nursing, medicine, and psychology demonstrates the articulation of complementary competencies required for a comprehensive and intersectoral approach to the health of populations experiencing homelessness.

The length of professional experience emerges in this study as a relevant element for understanding the practices developed within the service. PS1 and PS4, with eleven years of experience, represent professionals with a consolidated trajectory in expanded primary care. Prolonged engagement in the CnR contributes to the strengthening of care bonds, recognition of territorial dynamics, and improvement of longitudinal care processes [22]. Conversely, the presence of more recently incorporated professionals, such as PS2 and PS3, indicates an institutional process of team renewal and recomposition, introducing updated perspectives and fostering innovative strategies [23]. The ethical–political dimension of this work requires not only technical competence but also the capacity to recognize and respond to multiple forms of vulnerability [24].

The synthesis presented in Table 2 demonstrates that the challenges faced within the CnR are not isolated or circumstantial, but reflect structural tensions that affect both the living conditions of the population served and the organization of the service itself.

In this context, health vulnerability refers to conditions that increase or reduce the likelihood of illness [2]. Among individuals experiencing homelessness, this vulnerability is expressed through exposure to multiple and changing health risks, including tuberculosis, sexually transmitted infections, HIV/AIDS, hepatitis, chronic skin infections, leprosy, asthma, bronchitis, non-communicable chronic diseases, and frequent oral health needs [25,26]. International evidence reinforces the severity of this situation, showing that people experiencing homelessness have a twofold risk of all-cause mortality compared with the general population [27].

With specific regard to the axis of social vulnerability, the complexity of the work carried out by CnR teams requires them to act not only as healthcare providers but also as mediators of rights and coordinators of public policies that often fail to integrate spontaneously [28]. From an ethical–clinical perspective, the CnR is deeply grounded in the harm reduction paradigm, particularly in the management of alcohol, crack cocaine, and other drug use [29], promoting HIV/AIDS prevention and reducing vulnerabilities to other sexually transmitted infections.

The insecurity experienced during outreach activities cannot be reduced to an individual perception of risk. Rather, it should be interpreted, as proposed by Achille Mbembe [30], within the framework of the necropolitical governance of urban spaces, where certain populations are recurrently treated as disposable. The statement by PS1, “we find ourselves not knowing what to do, without support,” illustrates this point and expresses a sense of abandonment that compromises both physical safety and the institutional legitimacy of the service.

This insecurity is directly linked to the material precariousness that permeates the daily operation of the service. When PS2 states that “we often have to improvise” she makes explicit what Ayres [31] conceptualizes as the tension between the comprehensiveness of care and the actual conditions of work. The absence of basic supplies, adequate transportation, and minimal physical infrastructure hinders the consolidation of care bonds, destabilizes continuity of care, and weakens interdisciplinarity.

PS3’s observation—“management changes and everything changes”—not only synthesizes the everyday perception of instability but also directly resonates with what Merhy [32] describes as programmatic arrangements—fragile and dependent on shifting political will. When PS3 acknowledges that “everything changes,” he reveals the difficulty of planning long-term territorial actions and the impossibility of consolidating continuous training processes.

The subjective repercussions of this context become evident in PS4’s statement: “there are days when we come back completely exhausted.” This expression conveys, in stark terms, the ethical suffering described by Dejours [33], marked by the gap between what workers aspire to provide and what they are able to deliver under institutional constraints. França et al. [34] reinforce this point by demonstrating that, when teams lack clinical supervision, psychological support, or collective care spaces, everyday work tends to become a terrain of persistent strain.

Nevertheless, the narratives indicate that, even in the face of such limitations, professionals produce forms of care that approximate what Biehl and Petryna [35] term “care in worlds of abandonment.” Strategies such as attentive listening, mediation across service networks, gradual engagement, and the continuous reorganization of work constitute what authors such as Merhy [32] describe as micropolitical practices—ways of confronting, through embodied and relational actions, the structural failures of the state.

**Figure 1:**
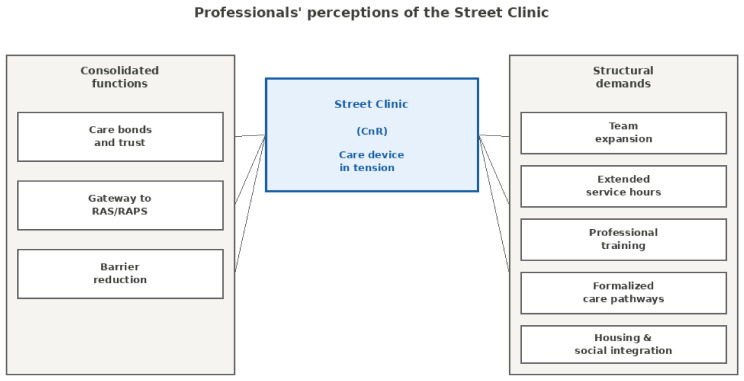
Thematic dimensions of professionals’ perceptions of the Street Clinic. The diagram illustrates the tension between consolidated functions effectively enacted in daily practice (**left**) and structural demands insufficiently addressed (**right**), with the Street Clinic positioned as a care device operating within this dynamic tension.

Figure 1 synthesizes professionals’ perceptions of the Street Clinic in Maringá into two complementary dimensions, highlighting the tension between what the service effectively accomplishes and what structural limitations still prevent it from achieving.

Beyond the visual organization of these dimensions, the findings indicate that the Street Clinic is perceived simultaneously as an effective point of access to care and as a fragile institutional arrangement marked by structural constraints. On the one hand, professionals recognize the service’s capacity to establish care bonds, facilitate entry into the Health Care Network (RAS), and reduce barriers to access. On the other hand, they emphasize persistent demands that remain insufficiently addressed, including the expansion of teams and service hours, ongoing professional training, the formalization of care pathways, and stronger integration with housing and social assistance policies.

The findings indicate that the coordinating role of the CnR is not only prescribed in normative documents but is effectively enacted in the daily practices reported by professionals. Territorial engagement, team mobility, active outreach, and the gradual approach to users constitute fundamental mechanisms for building trust and mediating between street contexts and services within the RAS and the Psychosocial Care Network (RAPS) [36,37,38]. Coordination with housing policies, social assistance, and rights protection emerges consistently both in the interviews and in the reviewed literature. Initiatives such as Housing First demonstrate that housing stability is a structural condition for therapeutic adherence and for the organization of life projects [39,40].

## 4. Conclusions

This study revealed that professionals working in the Street Clinic in Maringá experience a persistent tension between their ethical commitment to comprehensive care and the structural limitations that shape their daily work. This tension manifests as ethical suffering, not as individual fragility, but as a response to the mismatch between what the service requires and what the State provides in terms of infrastructure, institutional stability, and team support. At the same time, the narratives indicate that this suffering coexists with creative forms of resistance: micropolitical practices of bonding, listening, and presence in the territory that sustain care even when institutional conditions are insufficient.

The findings indicate that strengthening the Street Clinic cannot be achieved through isolated adjustments in funding or the mere expansion of teams, although such measures are necessary. What the narratives reveal is a more structural demand: the need for the CnR to be recognized as a state policy, with institutionalized care pathways, stable employment arrangements, continuous emotional support for teams, and effective coordination with housing and social assistance policies. Without such recognition, street-based work will continue to rely on the individual commitment of professionals to be sustained, which is ethically unsustainable in the long term.

This study presents limitations that must be acknowledged. The small number of participants and the focus on a single municipality preclude generalization to other contexts. The findings reflect the specific reality of Maringá and may differ from other municipal configurations. Nevertheless, the study contributes to the national debate by documenting, from the perspective of those working in street-based care, the contradictions that structure the CnR—between its potential as a care device and the precarious conditions under which it operates—thus reaffirming the urgency of policies that also care for the caregivers themselves.

## Figures and Tables

**Table 1:** Sociodemographic characterization of participants.

Participant	Academic Background	Area of Practice	Length of Service
PS1	Nursing Technician	CnR and private hospital	11 years
PS2	Nurse	CnR	1 year
PS3	Physician	CnR	2 years
PS4	Psychologist	CnR	11 years

Note: According to Ordinance GM/MS No. 1255/2021, CnR teams are normatively authorized to include Community Health Workers; however, this position was not included in the team composition at the time of the study.

**Table 2:** Reported Daily Challenges of Professionals Working in the Street Clinic in Maringá.

Thematic Axis	Identified Challenges	Strategies or Coping Mechanisms
Social vulnerability	Social stigma, violence, lack of housing and food, absence of identification documents	Active listening, gradual engagement, coordinated actions with CRAS, SUAS, Centro POP, and SAS
Bond with users	Difficulty in initial contact, distrust, refusal of care, situations involving substance use	Respect for users’ timing, gentle approach, use of supplies such as water/food, mediation by already engaged users
Infrastructure and resources	Deteriorated vehicle, difficulties accessing diagnostic tests, lack of appropriate spaces for certain procedures	Biweekly inventory control, adaptation of strategies, rational use of materials, referral to Primary Health Units (UBS)
Human resources and organization	Reduced team for city-wide coverage; workload burden and limited working hours (07:00–13:00)	Intersectoral coordination, focus on multidisciplinary work, collective prioritization, schedule reorganization
Communication with users	Cultural barriers, users’ preference for exclusively medical care, disagreements in triage	Explanations about the service’s role, reinforcement of teamwork, respect for users’ choices
Therapeutic planning	Difficulty adhering to therapeutic plans due to substance use and social instability; challenges in developing individualized Therapeutic Projects (PTS)	Adaptation of plans to users’ realities; prioritization of long-term bonds; ongoing health education
Notable/negative situations	Grief due to loss of users, frustration with care limitations, disrespect toward the homeless population	Team sharing, attention to professionals’ mental health, valuing achievements, active listening among colleagues

Note: The thematic axes and their respective challenges and strategies were constructed inductively from the hermeneutic analysis of the interviews, without a priori categories, and do not represent statistical frequency. PTS: Singular Therapeutic Project (Projeto Terapêutico Singular), a multiprofessional care-planning tool used in the Brazilian Unified Health System (SUS).

## Data Availability

The data presented in this study are available upon request from the corresponding author, due to ethical and privacy restrictions involving the research participants.

## References

[1] Almeida R., Araújo R.L. (2025). A questão social e a cidade: Contradições urbanas no neoliberalismo. Serviço Soc. Em Debate.

[2] Ayres J.R.C.M., França Júnior I., Calazans G.J., Saletti Filho H.C., Czeresnia D., Freitas C.M. (2003). O conceito de vulnerabilidade e as práticas de saúde. Promoção da Saúde: Conceitos, Reflexões, Tendências.

[3] Sicari A.A., Zanella A.V. (2018). Pessoas em situação de rua no Brasil: Revisão sistemática. Psicol. Ciência E Profissão.

[4] Silva V.G.P., Koshita L.H., Marques F.H., Silva T.M.G. (2024). Characterization of social conditions of homeless women in Maringá-PR. Int. J. Educ. Res..

[5] Meyer B.D., Wyse A., Logani I. (2023). Life and death at the margins of society: The mortality of the US homeless population. NBER Working Paper No. 31843.

[6] U.S. Department of Housing and Urban Development (HUD) (2024). The 2024 Annual Homelessness Assessment Report (AHAR) to Congress.

[7] Cleynen E., Ingelbeen B., Lenormand A., Kerami J., Nöstlinger C. (2025). Mortality and barriers to healthcare among people experiencing homelessness in Paris: A mixed-methods study. Int. J. Equity Health.

[8] Natalino M. (2022). Estimativa da População em Situação de rua no Brasil (2012–2022).

[9] Agência Brasil Mais de 335 mil Pessoas Vivem em Situação de rua no Brasil. https://agenciabrasil.ebc.com.br/direitos-humanos/noticia/2025-04/mais-de-335-mil-pessoas-vivem-em-situacao-de-rua-no-brasil.

[10] Travis P.B., Samantha S.L., Stephen W.H. (2018). Cardiovascular disease and homelessness. J. Am. Coll. Cardiol..

[11] Brasil. Ministério da Saúde (2025). Diretrizes para Organização e Funcionamento das Equipes de Consultório na Rua.

[12] Timóteo A.V.G., Silva J.V.D.S., Gomes L.K.G., Alves A.S.S., Barbosa V.M.D.S., Brandão T.M. (2020). Caracterização do trabalho e ações desenvolvidas pelas equipes do Consultório na Rua de Maceió-AL. Enferm. Em Foco.

[13] Oliveira R.G. (2018). Práticas de saúde em contextos de vulnerabilização e negligência. Saúde E Soc..

[14] dos Santos Silva J.V., dos Santos Júnior C.J., Bezerra W.C., Brandão T.M. (2021). Consultório na Rua: Experiências e sentimentos vivenciados pelos profissionais. Medicina.

[15] Hallais J.A.S., Barros N.F. (2015). Consultório na Rua: Visibilidades, invisibilidades e hipervisibilidade. Cad. Saúde Pública.

[16] Carneiro A. (2017). Saúde Mental e População em Situação de Rua.

[17] Gadamer H.G. (1997). Verdade e Método: Traços Fundamentais de uma Hermenêutica Filosófica.

[18] Silva N.E.K., Ayres J.R.C.M. (2024). Contar histórias, cuidar da vida: Educação em saúde baseada em narrativas. Physis.

[19] Minayo M.C.S. (2017). Cientificidade, generalização e divulgação de estudos qualitativos. Ciência Saúde Coletiva.

[20] Fontanella B.J.B., Luchesi B.M., Saidel M.G.B., Ricas J., Turato E.R., Melo D.G. (2011). Amostragem em pesquisas qualitativas: Proposta de procedimentos para constatar saturação teórica. Cad. Saúde Pública.

[21] Brasil. Ministério da Saúde (2021). Portaria GM/MS No. 1.255, de 18 de junho de 2021. Dispõe Sobre as Diretrizes de Organização e Funcionamento das Equipes de Consultório na Rua e os Critérios de Cálculo do Número Máximo de Equipes de Consultório na Rua, por Município e Distrito Federal, por meio da Alteração da Portaria de Consolidação GM/MS No. 2, de 28 de Setembro de 2017.

[22] Carvalho C.P.P.X., Terra L.F., Barros Souza B. (2024). O Estado e a saúde de populações vulneráveis. Cuad. Educ. Desarro..

[23] Martins A.L.J., Souza A.A.D., Fernandes L.D.M.M., Oliveira A.M.C., Cordeiro J.C., Oliveira A.F.D., Magalhães Júnior H.M. (2023). A interface entre as políticas públicas para a população em situação de rua. Ciência Saúde Coletiva.

[24] Paiva I.K.S., Guimarães J. (2023). População em situação de rua e Rede de Atenção Psicossocial. Physis.

[25] Ximenes V.M., Malhado S.D.C.B., Moreno R.S., Monteiro M.N.B.P. (2021). Apoio social para pessoas em situação de rua. Psicoperspectivas.

[26] Machado T.G.D.O., Lawder J.A.C., Souza J.B.D., Matos M.A.D., Freire M.D.C.M. (2022). Condição periodontal de adultos em situação de rua. Ciência Saúde Coletiva.

[27] White J., Moriarty Y., Lau M., Cannings-John R., Palmer A., Weightman A.L., Kiseleva M., Batty G.D. (2025). Homelessness and risk of cause-specific mortality: A systematic review and meta-analysis. medRxiv.

[28] Veridiano A.L., Andrade L., Gomes A.H. (2017). Práticas intersetoriais na atenção às pessoas em situação de rua: Uma atuação entre “saúde” e “assistência social”. Rev. Visão Gestão Organ..

[29] Medeiros P.F.P.D., Rameh-de-Albuquerque R.C., Almeida R.B.F.D., Campos-Boulitreau A.R.L., Valois-Santos N.T., Marques A.L.M. (2023). Consultório de Rua: Cuidado no território na interface entre HIV/Aids, drogas e Redução de Danos. Saúde Em Debate.

[30] Mbembe A. (2018). Necropolítica.

[31] Ayres J.R.C.M. (2004). Cuidado: Trabalho e interação nas práticas de saúde. Interface.

[32] Merhy E.E. (2014). A clínica do trabalho vivo. Saúde Em Debate.

[33] Dejours C. (1992). A Loucura do Trabalho.

[34] França R.J., Dias J.M., Andrade C.S.S., Guimarães W.S., Campos I. (2025). Consultório na Rua: Uma análise do acesso à saúde pela população em situação de rua a partir do número de equipes e dos desafios enfrentados. RIES.

[35] Biehl J., Petryna A. (2013). Cuidado: Histórias de Violência e Redenção.

[36] Vargas E.R., Macerata I. (2018). Contribuições das equipes de Consultório na Rua para o cuidado e a gestão da atenção básica. Rev. Panam. Salud Publica.

[37] Araujo A.C.C., Pires R.R. (2018). Redução de danos na Atenção Psicossocial: Concepções e vivências de profissionais em um CAPS Ad. Tempus.

[38] Almeida P.F., Medina M.G., Fausto M.C.R., Giovanella L., Bousquat A., Mendonça M.H.M. (2018). Coordenação do cuidado e atenção primária à saúde no Sistema Único de Saúde. Saúde Debate.

[39] Brasil. Ministério dos Direitos Humanos e da Cidadania (2023). Plano Nacional Ruas Visíveis: Plano de Ação e Monitoramento para Efetivação da Política Nacional para a População em Situação de Rua.

[40] World Health Organization (2023). Housing First as a Public Health Strategy.

